# Supplementary material for: The causal relationship between the human gut microbiota and pyogenic arthritis: a Mendelian randomization study
Source: Front Cell Infect Microbiol. 2024 Nov 26;14:1452480. doi: 10.3389/fcimb.2024.1452480 (PMC11629706; doi:10.3389/fcimb.2024.1452480)
Supplement: Supplementary file 1 [file DataSheet1.zip › mendelian test/ebi-a-GCST90027589.csv_forest.pdf]

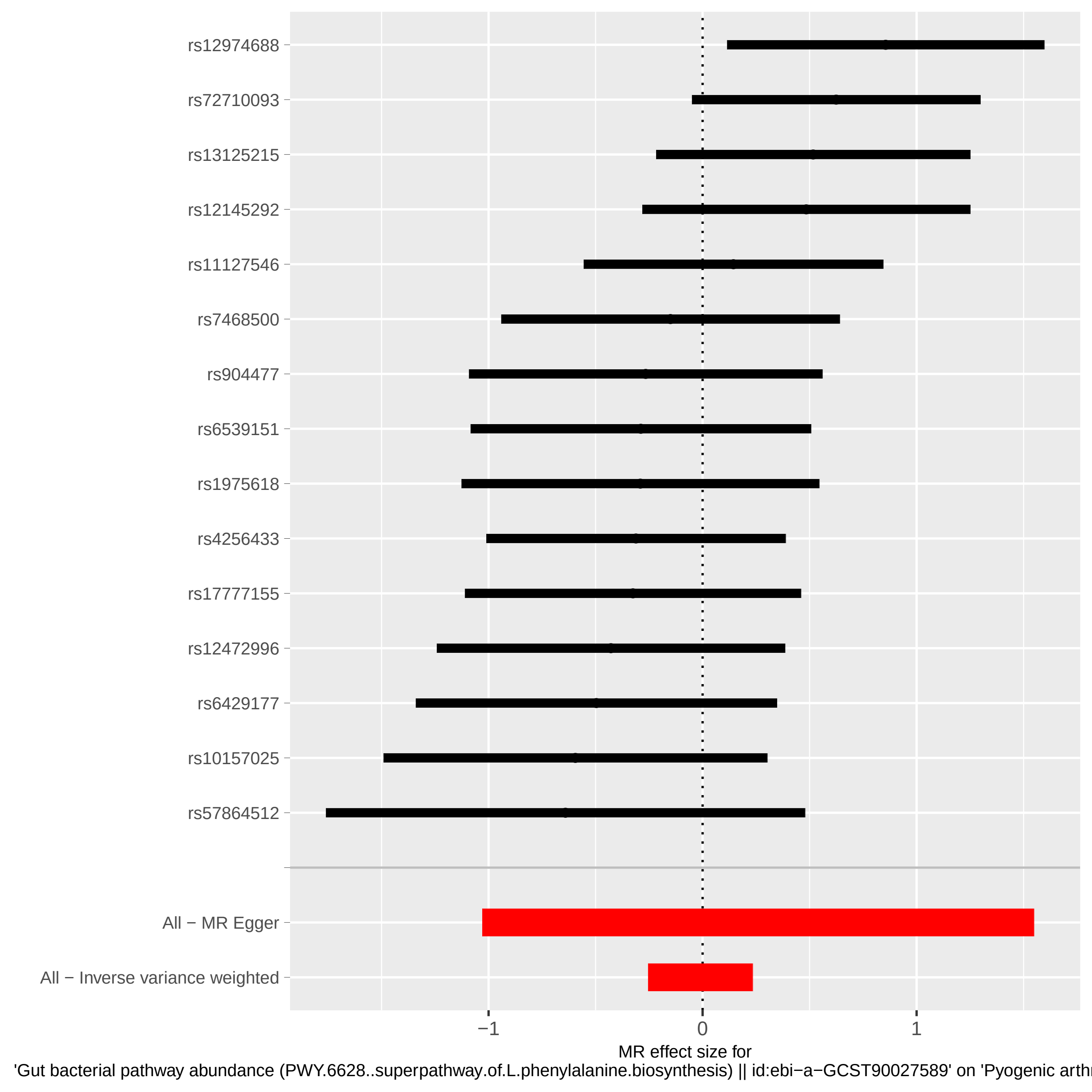
rs12974688
rs72710093
rs13125215
rs12145292
rs11127546
rs7468500
rs904477
rs6539151
rs1975618
rs4256433
rs17777155
rs12472996
rs6429177
rs10157025
rs57864512
All − MR Egger
All − Inverse variance weighted
−1
0
1
MR effect size for
'Gut bacterial pathway abundance (PWY.6628..superpathway.of.L.phenylalanine.biosynthesis) || id:ebi−a−GCST90027589' on 'Pyogenic arth
